# The gender-based violence and recovery centre at Coast Provincial General Hospital, Mombasa, Kenya: An integrated care model for survivors of sexual violence

**DOI:** 10.1371/journal.pmed.1002886

**Published:** 2019-08-02

**Authors:** Marleen Temmerman, Emilomo Ogbe, Griffins Manguro, Iqbal Khandwalla, Mary Thiongo, Kishor N. Mandaliya, Lou Dierick, Markus MacGill, Peter Gichangi

**Affiliations:** 1 International Centre for Reproductive Health, Mombasa, Kenya; 2 International Centre for Reproductive Health, Ghent University, Ghent, Belgium; 3 Aga Khan University, Nairobi, Kenya; 4 Coast Provincial General Hospital, Mombasa, Kenya; 5 Green Ink medical editing, Edinburgh, Scotland, United Kingdom; 6 University of Nairobi, Nairobi, Kenya

## Abstract

Marleen Temmerman and colleagues describe a model of care for people who have experienced sexual violence, set in Kenya.

**Summary points**

- Sexual violence (SV) is highly prevalent and a major public health problem globally. In Kenya, an estimated 32% of females and 18% of males were reported to have experienced SV before the age of 18 years.
- This paper presents a data set collected between 2007 and 2018 and describes the gender-based violence and recovery centre (GBVRC) model under which survivors of SV were cared for at a 24-hour public hospital in Mombasa, Kenya—including its development, implementation, achievements, and challenges.
- The GBVRC model is a partnership that provides (in addition to emergency healthcare) mental health support, paralegal services, and integrated cooperation with police, judiciary, local leaders, and the wider community. The Mombasa GBVRC has provided post-SV care to 6,575 people reporting SV, of whom 88% were female and over 50% were younger than 16 years. Over 90% of the perpetrators were family, neighbours, community members, or in some other way known to the survivors.
- The low rate (19%) of attendance by survivors for the second counselling visit suggests a more robust strategy is needed for follow-up—for example, by referring people back to smaller, closer health facilities. A second limitation was a lack of trained staff, although this is an expected issue in sub-Saharan Africa. There was also a low rate of legal resolution to the cases. This may be due to the need for education about the standard of evidence required by courts.
- The experiences of successful and sustainable implementation of the GBVRC model should strengthen arguments for service delivery for people experiencing SV in this and similar settings.

## Introduction

Sexual violence (SV) is a major public health problem globally [1,2]. Although both sexes suffer SV, women are disproportionately affected [2]. This may reflect underlying harmful gender norms within many societies [3,4]. SV is associated with numerous adverse health outcomes, including HIV and other sexually transmitted infections (STIs), unintended pregnancy and unsafe abortions, and miscarriage, preterm birth, and stillbirth [3,5–7]. SV is also associated with mental health outcomes, including posttraumatic stress disorder, anxiety and depression, and an increased risk of ideated or attempted suicide [8–10].

The availability and accessibility of quality post-SV care is a challenge in all settings, but especially in low- and middle-income countries. In many such settings, management of SV is focused on physical injuries, STIs, and pregnancy [8]. Longer-term follow-up with psychosocial counselling and legal support is suboptimal, and the strengthened integration of these services is needed to improve overall health and life outcomes [11–13]. Although uptake of post-SV services has improved recently, rates of reporting to the police and legal follow-up of cases is about 7% globally [14].

Gender-based violence centres hold great potential to improve not only the health but also the legal follow-up and outcomes of SV survivors by strengthening referrals to the police and other legal channels and by collecting good-quality forensic specimens [15,16].

### The Kenya context

Studies on SV in Kenya and other African countries estimate a high prevalence among young boys and girls. A multicountry survey conducted in 2010 reported that, in Kenya, 32% of females and 18% of males experienced SV before the age of 18 years [17]. In the other six countries in the study, SV prevalence ranged from 4.4% among females in Cambodia to 37.5% among females in Eswatini. A similar, cross-sectional household survey conducted among young women aged 15–24 years in Kenya and Zambia reported overall SV rates of 21.4% among females under the age of 18 years [18]. A number of progressive national policies to address SV have been instituted in Kenya. However, implementation challenges such as a lack of comprehensive community structures for long-term follow-up, inadequate health-facility infrastructure for comprehensive SV services, poorly collected forensics, and poor linkage between health and legal sectors [19,20] remain barriers to effective SV response.

### Models of SV care

Different models for delivering post-rape care exist in Kenya. The three main models are as follows: healthcare centres and outpatient clinics; integrated care within hospitals giving day and night service every day of the week; and one-stop, stand-alone centres [12,20]. Health centres or outpatient clinics, public or private, are close to the community and offer survivors basic emergency care without additional services such as laboratory and psychosocial support or specialist care. Most are open for limited hours and refer for further management [10]. If a good network is established, they can improve access to an intersectoral network of services, including legal support. This is, however, difficult to achieve in low-resourced rural areas because the number of providers (clinical, psychological, community, and legal) is limited across all sectors. A bigger disadvantage is that, in small communities, providers are members of the community, and confidentiality and providers’ fear of retaliation by alleged perpetrators may be a challenge [10]. One-stop centres provide comprehensive medical, legal, and psychosocial services, but the stigma of SV and the limited availability at these stand-alone centres may reduce uptake by survivors. If they are run by the government, they may also draw staff from other health facilities, yet they deal with a small number of survivors, bringing into question their cost-effectiveness. Integrated services centres within public or private hospitals offer the potential to provide comprehensive SV services around the clock and make surgical services, antiretroviral therapy (ART), counselling, and other specialised treatment available. They also serve as SV referral centres for smaller facilities because they are open for 24 hours. They are likely to provide services to a larger number of survivors, and referrals from other hospital departments are easier than at stand-alone sites [10].

## The gender-based violence and recovery centre model

The gender-based violence and recovery centre (GBVRC) is an integrated post-SV service–delivery model within a government referral facility, the Coast Provincial General Hospital (CPGH), in Mombasa, Kenya. CPGH is the second-largest government hospital in Kenya, with a 700-bed capacity. It serves as the tertiary referral centre for the entire coastal region, with an estimated catchment population of 3.5 million people. We describe its development, implementation, achievements, and challenges and present a data set from several thousand survivors using it.

This model is integrated in the sense, first, that services are provided within a section of the hospital’s outpatient department and referrals are made to and from other departments as necessary. Second, in addition to emergency clinical care provided to SV survivors, mental health support, paralegal services and links with police, judiciary, local leaders, and the wider community are also availed to survivors, and all of these are coordinated at the centre using established standard operating procedures. Third, clinical staff hired by the International Centre for Reproductive Health (ICRH) Kenya are seconded to work at the GBVRC, which is run by the CPGH.

The GBVRC was set up at the CPGH in 2007 as a collaboration between the Ministry of Health (MoH) and ICRH Kenya, a nongovernmental sexual and reproductive health research organisation founded in 2000. CPGH is the second-largest public hospital in Kenya, a country with a population of over 50 million.

The main aim of the GBVRC is to improve SV management, demonstrate the feasibility of establishing a comprehensive, multisectoral care centre within a public health facility in Kenya, and provide a learning site for best practice. We aim to improve SV management by providing an acceptable model in SV care that would increase the number of survivors who seek care. We also aim to improve the clinical and psychological outcomes of SV by preventing HIV infections, unintended pregnancies, STIs, and psychological disorders such as depression and anxiety. We also aim to increase the number of SV cases reported to the police and followed up in court.

To ensure it was user centred and sustainable, the GBVRC model was designed in consultation. Engagement meetings were held with community representatives, police, lawyers, social workers, and the MoH to identify gaps and develop a model that integrated medical and legal services, with strong community participation. An advisory board was then established to supervise implementation and institute any refinements needed. This board was composed of all SV stakeholders within the county, health facility directors, administrators, clinical staff, local leaders, community members, police, and judiciary. A structure for continual supervisory support and mentorship was also created.

Every month, the GBVRC staff hold meetings with representatives from key hospital departments to review each case treated during the month, identify gaps in management and the referral process, and implement quality-improvement measures. Hospital departments represented during these meetings include outpatients, accident and emergency, laboratory, pharmacy, social services, administration, surgery, obstetrics and gynaecology, paediatrics, and mental health. Every 3 months, the centre holds a users’ committee composed of health facility staff, the police, and the department of the public prosecutor. These meetings review cases presented to court and discuss challenges with specimen collection, police preparation of cases, and prosecution to improve outcomes. A larger, countywide, multisectoral stakeholder meeting is held quarterly to promote collaboration in preventing SV within the community.

People reporting SV are either self-referred, referred from police stations, or referred from other health facilities. Because of the long-standing partnerships, clear guidance at the point of referral, and the centre’s reputation in the region, police referrals and referrals from health facilities proceed straight to the centre once they get to the hospital. Police referrals are often accompanied by a gender desk police officer, especially for minors. Some self-referrals are first seen at the outpatient department, especially if they do not report SV as the chief complaint. However, clinical staff in outpatients are trained to probe for pointers towards a history of SV. For cases in which they suspect SV, they offer basic medical care as well as treatment for physical injuries according to clinical guidelines and protocols, and then they refer the survivors to the GBVRC. All services are provided without charge.

When a survivor of SV declines immediate examination because of trauma or discomfort, they receive medical care and counselling. After obtaining informed consent, the SV nurse collects data on the survivor’s sociodemographics, the circumstances of the incident, and the perpetrator’s characteristics using a standardised MoH post-rape care form, including for psychological assessment (MoH 363). (Incident type is classified based on definitions in the Sexual Offences Act of Kenya 2006.) Data collected using the forms are entered contemporaneously into a secure database developed by ICRH and are used to generate reports, conduct research, and provide evidence-based feedback to all stakeholders. Ethical approval for data use and publication was obtained from the African Medical and Research Foundation (AMREF) Ethics Review Committee in Kenya.

Pregnancy, HIV, and other STIs are explained and tested for at the GBVRC. Rapid HIV test kits are used. When the survivor tests negative, HIV postexposure prophylaxis (PEP) is provided. Survivors who test positive during the initial visit or who seroconvert are referred for immediate ART at the HIV comprehensive care clinic within the hospital. STI prophylactic treatment and emergency contraception are also offered to eligible survivors. Following clinical management, survivors receive trauma counselling and psychosocial support. Based on the national guidelines, each survivor should undergo five counselling sessions, at 2-week intervals. Subsequent sessions can either be one-on-one or group. When necessary, the counsellor visits the survivor for home counselling. Counsellors employed at the GBVRC have training in nursing or clinical medicine and must be accredited by the Kenya Association of Professional Counsellors or recognised as SV trauma counsellors by Public Health and Sanitation or the Ministry of Medical Services. They also undergo continuous professional development on providing counselling services to minors.

After medical and psychological care, survivors who present directly to the hospital and have not reported to the police must be referred to the nearest station. They are accompanied by a paralegal if requested. If the survivor is a minor, the GBVRC counsellor will call for the police to come to the centre; this is also the practice if there is suspicion that the person accompanying may be the perpetrator. This process is enabled by the positive engagement and relations with the police gender desks in the county.

**Fig 1:**
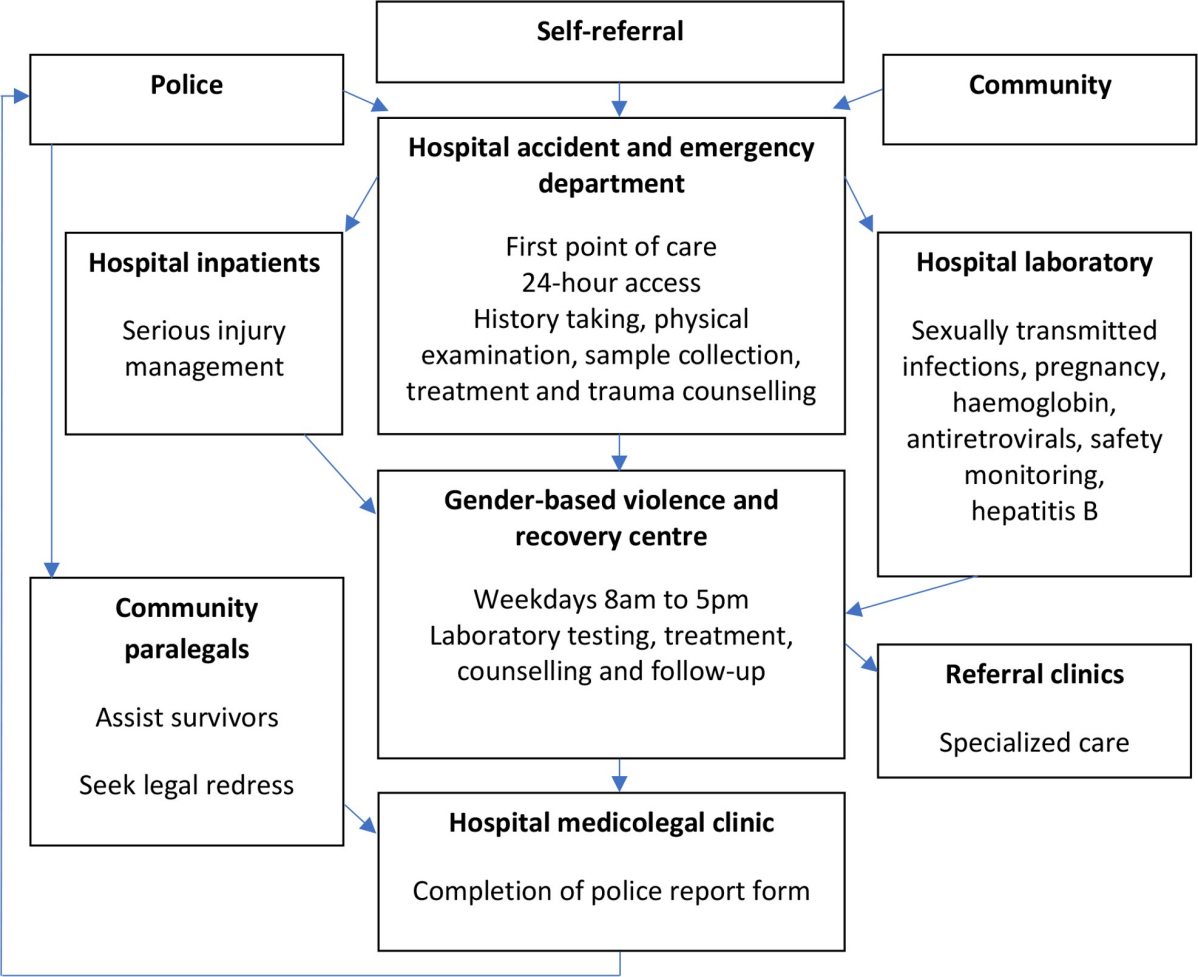
The Coast Provincial General Hospital service–delivery model and referral pathways for survivors of sexual violence.

Fig 1 summarises the GBVRC model of care in operation at CPGH.

The paralegals are recruited from the community to provide legal advice, counselling, and court preparation. They undergo an intensive 6-month training in SV and on the relevant basic laws and procedures based on a national curriculum. Working voluntarily, with a modest stipend for transport, they serve as focal persons for community education. They conduct community dialogues to educate the community, identify SV survivors, and engage them to seek post-SV services and report to the police. They also follow up with survivors to determine outcomes after counselling and ensure they are safe within their environment, given that, in most cases, the perpetrators belong to the same environment as the survivors.

A combination of national and international sources, detailed in the Financial Disclosure, have provided initial and ongoing funding to the GBVRC. Initial funding to the centre through ICRH Kenya was from the Danish International Development Agency (DANIDA). At the end of this funding, the centre was supported through the United Nations Population Fund (UNFPA) Kenya office and from individual contributions.

## Findings of the GBVRC model in action

The GBVRC has operated from 2007 to date using the model described previously. Between 2007 and December 2018, 6,575 survivors of SV have received clinical, psychosocial, and legal support. Three full-time counsellors and 35 paralegals are engaged by ICRH Kenya to provide services during the day. CPGH assigns staff from the outpatient department to SV care at night and during weekends and provides other services such as surgical care and ART free of charge.

### Baseline social and demographic characteristics

Since 2007, the GBVRC has attended to 6,575 individual SV survivors. Table 1 presents survivor characteristics and incident details. Most survivors were female (5,837 or 88%), and over half were below 16 years of age (3,670 or 56%). Male survivors were often younger—median age, 9 years, interquartile range (IQR), 6–13 years, compared with a median age of 14 years for females, with an IQR of 9–17 years. Indeed, among males, more than half the survivors were under 11 years old (402 or 54%), compared with about a quarter of females (1,566 or 27%).

**Table 1:** Characteristics of SV survivors and their management at the GBVRC in Mombasa, Kenya (2007–2018).

	Overall	Females	Males
	*n* = 6,575	*n* = 5,837, 88%	*n* = 738, 12%
**Survivor median age**	14 years (8–17 IQR)	14 years (9–17 IQR)	9 years (6–13 IQR)
**Perpetrator median age**	28 years (20–40 IQR)	29 years (20–40 IQR)	25 years (17–40 IQR)
**Survivor age group**	**Number (%)**	**Number (%)**	**Number (%)**
≤10 years	1,968 (30)	1,566 (27)	402 (54)
11–15 years	1,702 (26)	1,496 (26)	206 (27)
16–20 years	1,970 (30)	1,906 (33)	64 (9)
>20 years	935 (14)	869 (15)	669 (9)
Perpetrator* **known to survivor**	5,604 (85)	4,949 (85)	655 (89)
**Type of assault**			
Vaginal	5,114 (80)	5,076 (89)	–
Anal	1,037 (16)	427 (7)	610 (92)
Oral	165 (3)	133 (2)	32 (5)
Other	84 (1)	68 (1)	16 (3)
**Condom used by perpetrator**	344 (3)	321 (6)	23 (3)
**Survivor response**			
Reported to the police	3,334 (51)	2,961 (51)	373 (52)
Attended a health facility immediately	3,026 (48)	2,697 (47)	329 (46)
Changed clothes	5,285 (81)	4,979 (83)	640 (88)
Bathed	5,202 (81)	4,586 (80)	616 (85)
**Clinical management**			
PEP given	3,115 (48)	2,766 (48)	349 (48)
Emergency contraceptive given	–	3,117 (61)	–
STI treatment given	3,366 (52)	2,996 (53)	370 (51)

* Data were available on perpetrators for 6,352 cases of SV (96%).

Abbreviations: GBVRC, gender-based violence and recovery centre; IQR, interquartile range; PEP, HIV postexposure prophylaxis; STI, sexually transmitted infection; SV, sexual violence.

In almost nine out of 10 cases, the SV perpetrator was known to the survivor (whether as an intimate partner or family relation, or as a member of the neighbourhood community). When data were available (6,352 or 96%), the estimated age of the perpetrator was between 20 and 40 years; perpetrators against males were generally younger than against females, although this difference was not significant (*p* = 0.58). Women most commonly suffered vaginal assault (5,076 or 89%) and males, anal assault (610 or 92%). In 97% of all cases, the perpetrator’s assault was without a condom. Most (81%, 5,202) of the survivors had either bathed or changed their clothes immediately after the incident.

### HIV and STIs and family planning

In total, 98% of survivors received HIV counselling and testing at the initial visit. Among those tested, 98% tested HIV negative, 48% (3,115) of whom received PEP. All those who tested positive were referred for immediate ART, although we do not have data on the proportion successfully linked. Prophylactic STI treatment was provided to 52% of the SV survivors (3,366), and among women who were vaginally assaulted, 3,117 (61%) received emergency contraceptive pills. Follow-up data on seroconversions, pregnancies, and STI infections were unavailable.

### Sources of referral

Comprehensive data on referral sources were available from 2013, when referral source data were included in the data-collection forms. In the 5 years between 2013 and 2018, 75% of the survivors who received care were referred from a police station. The remaining proportion were either self-referred or referred from peripheral health facilities. There was no significant change in the proportion referred from the police over the years. Data on referrals to the GBVRC for each year are presented in Table 2.

**Table 2:** Sources of survivor referrals to the GBVRC from 2013 to 2018.

Year	Police	Self-referral	Other facilities	Proportion from the police (%)
2013	480	45	86	79
2014	528	122	50	75
2015	590	173	32	74
2016	520	144	39	74
2017	432	73	55	77
2018	500	108	47	76

Abbreviation: GBVRC, gender-based violence and recovery centre

### Follow-up visits for psychological care

Data on survivor follow-up were available from 2013, after revision of the data-collection tool. Of 3,973 survivors who received care in these 5 years, 19% (746) came back for the second counselling visit, and 10.8% came for the third. Only 57 (1.4%) came for all five visits. Data on the proportion seen during each follow-up visit are presented in Table 3. Age- and sex-specific follow-up data were unavailable.

**Table 3:** Number and proportion of sexual violence survivors returning for follow-up.

Year	Initial visit	Visit 2	Visit 3	Visit 4	Visit 5
2018	691	147	73	35	9
2017	573	87	71	20	3
2016	696	138	71	99	16
2015	759	116	82	22	8
2014	638	111	58	10	4
2013	616	147	76	29	17
Total	3,973	746	431	215	57
Percentage	100	18.8	10.8	5.4	1.4

### Legal support and follow-up

Data on the follow-up of cases in court (Table 4) were available from 2015, when the centre started recording these data. Between 2015 and 2018, 2,719 survivors received care and were referred for legal services. Of these cases, 360 progressed to court, and 31 convictions have been secured, plus three acquittals, whereas all other cases are ongoing. Perpetrator sentences were between 9 years and life imprisonment.

**Table 4:** Legal outcome of sexual violence case referrals.

Year	Number of cases who received care at the GBVRC	Cases that ended in court	Outcomes
2015	759	113	3 convictions
2016	696	91	6 convictions
2017	573	62	13 convictions and 1 acquittal
2018	691	94	9 convictions and 2 acquittals

Abbreviation: GBVRC, gender-based violence and recovery centre

## Discussion

We have described the design and operation of the GBVRC based in Mombasa, Kenya, which provides comprehensive care to survivors of SV and serves as a learning centre to the region and the country. From 10-year follow-up data, 88% of survivors are female, with an overall median age of 14 years, although male survivors are significantly younger. In 85% of cases, the perpetrators are known to the survivors, and over 75% of survivors are referred from the police stations. Follow-up for subsequent trauma counselling is suboptimal, and only 19% came back for subsequent counselling after the initial session. Based on data from 2015 to 2018, 13% of cases seen at the centre were successfully presented to court, with 31 convictions to date.

These data highlight the need to tailor SV services to a generally young population. The age and sex distribution of SV and the details surrounding the incidents reported at our centre mirror reports from other studies and programmes within the country [20], and in other settings [21,22], and suggest similar patterns of vulnerability in most communities. Importantly, whereas males accounted for a small proportion overall—12% of cases—and similar studies have also reported a lower proportion of male survivors compared with females [23,24], over half were aged 11 years and below. This underscores the very high, yet often underestimated, vulnerability to SV of very young male children. Indeed, some studies suggest that SV among males is increasing [25].

Our findings emphasise that most perpetrators are close to the survivors. This has also been reported in other studies [21,26]. One study reported up to 30% of perpetrators being either a parent or stepparent [27]. At times, the perpetrators may be the persons accompanying the survivors to care. GBVRC staff need to have a heightened awareness and a low threshold for suspicion for this. If necessary, social or child protection services should be engaged at the earliest opportunity. Continual community education should take account of the fact that most survivors bathe or change clothes immediately following an assault or report to the facility several days after the incident. Community dialogue sessions by paralegals remain a key priority to improve overall knowledge on SV management within the community, including the steps needed after an incident.

It is worth noting that 75% of survivors who received care were referred from a police station. Others were referred from other facilities for psychosocial and legal support. Given that 6,575 survivors presented under the GBVRC model at this hospital and almost all consented to integrated care, we believe that such a model—providing comprehensive clinical, psychosocial, and legal support within a public health facility and pairing it with strong community engagement—may increase the uptake of SV services and improve the reporting of cases. Equally important to overall uptake is that populations such as men, who may find it difficult to seek treatment in stand-alone SV facilities, may be helped by the reduced stigma risk of using a centre that is nested within a public facility.

Among survivors treated between 2015 and 2018, only 13% of cases went to trial. The rest did not proceed, most likely because survivors and their families resolved out of court or there was inadequate evidence for prosecution, as the survivors may have changed clothes, had a bath, or reported several days after the incident. We could not confirm which of these was most plausible because of the sensitive nature of police records. However, similar rates for court follow-up and convictions (13%) have been reported in South Africa [28] and may highlight the sociocultural difficulties in legal follow-up, especially when families and close community members may be the perpetrators. Another key challenge was that although 45 paralegals received training, only 35 remain, as they work on a volunteer basis with a modest stipend. Important to note is that many of the cases are ongoing, and it often takes years for a case to be concluded; therefore, the final proportion of convictions among those prosecuted is likely to be much higher.

One key gap in the GBVRC model is the suboptimal follow-up of survivors after the first visit. Only 19% came back for the second counselling visit, and less than 2% returned for all five counselling sessions. One reason could be lack of transport or of motivation to visit the health facility, considering that most survivors are from low socioeconomic backgrounds. Counsellors could conduct home visits, but this is hardly achievable given the few counsellors available at the centre. Establishing a more robust strategy for follow-up by referring survivors back to smaller health facilities nearer to them is more plausible and would be achieved by training staff at these facilities to provide post-SV trauma counselling. A second challenge is the lack of adequately trained staff to provide comprehensive coverage 24 hours a day. This is expected; in most public health facilities in sub-Saharan Africa, staffing is hardly adequate to cover core hospital functions. Inadequate staffing may explain the low proportions of eligible survivors who received PEP or of females who received oral emergency contraceptives.

Such constraints can be addressed through multipronged strategies for health-system strengthening. Within GBVRC facilities, and in such referral hospitals as CPGH, all staff who are likely to work in the outpatients department should receive adequate core training plus regular refreshers on the comprehensive integrated management of SV. Clinical staff rotation around the whole hospital means that the core training should not be limited to certain departments. We would further recommend that SV training modules are integrated into medical and nursing curricula. Finally, community engagement is equally important: community health volunteers should be empowered to participate in the SV response and to complement, where they are available, paralegals—to improve SV awareness, knowledge, attitudes, and action within the community.

## Conclusion and the future of the GBVRC model

The GBVRC model demonstrates that good-quality, comprehensive SV services can be integrated sustainably into routine health services within public health facilities. It is a replicable model for such interventions in African countries and has the potential to increase the uptake of SV services, raising the proportion of survivors seeking care and/or reporting to the police. Designing and implementing such models requires multisectoral collaboration and continual community support. SV services in similar settings need to be tailored to the needs of generally young and predominantly female populations, although interventions targeting men and very young males should be scaled up.

## Abbreviations

ART: antiretroviral therapy
AMREF: African Medical and Research Foundation
CPGH: Coast Provincial General Hospital
DANIDA: Danish International Development Agency
GBVRC: gender-based violence and recovery centre
ICRH: International Centre for Reproductive Health
IQR: interquartile range
MoH: Ministry of Health
PEP: HIV postexposure prophylaxis
STI: sexually transmitted infection
SV: sexual violence
UNFPA: United Nations Population Fund
